# European vs. Egyptian HCV-4 Patients with Elevated Baseline HCV RNA, Treated with PEG-IFN-α2a and Ribavirin: The Role of Rapid and Early Virologic Response

**Published:** 2010-09-01

**Authors:** Dimitrios Dimitroulopoulos, Ioannis Elefsiniotis, Christos Pavlidis, Dimitrios Xinopoulos, Klisthenis Tsamakidis, Stamatina Patsavela, Dimitrios Kypreos, Ageliki Ferderigou, Dimitrios Korkolis, Sotirios Koutsounas, Georgios Saroglou, Emmanouil Paraskevas

**Affiliations:** 1 Department of Gastroenterology, Agios Savvas Hospital, Athens, Greece; 2 Department of Internal Medicine, Helena Venizelou Hospital – University of Athens, Athens, Greece; 3 Reference Center for Viral Hepatitis, IKA, Athens, Greece; 4 Department of Biochemistry, Agios Savvas Hospital, Athens, Greece; 5 First Surgical Department, Agios Savvas Hospital, Athens, Greece

**Keywords:** HCV, Genotype 4, EVR, RVR, Treatment

## Abstract

**Background and Aims:**

Despite the recent spread of hepatitis C virus genotype 4 (HCV-4) into European countries, very little is known about the influence of ethnicity on treatment outcomes in patients with HCV-4. The aim of this study was to compare the virologic response (VR) rates of: rapid virologic response (RVR), early virologic response (EVR), VR at 24 weeks of treatment, at end of treatment (EoT), and sustained virologic response (SVR) of European and Egyptian HCV-4 patients.

**Methods:**

Sixty (30 Europeans – Group A; and 30 Egyptians – Group B) chronic HCV-4 subtype A adult patients with elevated baseline viral load (>800 000 IU/m L) were treated for a fixed period of 48 weeks with pegylated interferon α2a (PEG-IFN- α2a) and ribavirin. During the study, HCV-RNA levels were measured at weeks 4,12,24,48 and 72.

**Results:**

Baseline characteristics, including liver histology, were similar in the two groups. RVR, EVR and HCV-RNA at week 24 in Groups A and B were (RVR 26.7% vs. 30.0%) (EVR 23.3% vs. 16.7%) (in week 24 13.3% vs. 16.7%). Overall SVR rates were 36.7% (11/30) for Group A and 26.7% (8/30) for Group B (P = 0.59). For group B, RVR was the weakest indicator for SVR as compared with RVR of group A, where RVR was the best SVR indicator

**Conclusions:**

The overall response to treatment was similar, but ethnic origin or previous history and treatment of schistosomiasis may influence intermediate response rates of chronic HCV-4a infected patients with elevated baseline HCV-RNA.

## Introduction

Hepatitis C virus (HCV) infection is a major health problem with an estimated 175 million infected people worldwide [1]. At least 6 major HCV genotypes have been identified, and each one differs from the others by 30%-35% of its nucleotide site sequence [1][2].

HCV genotype 4 (HCV-4) is common in the Middle East and in Africa, where it is responsible for more than 80% of HCV infections [3].

In Egypt, where hepatitis C is highly endemic, HCV-4 is the predominant subtype [3][4]. Recently, HCV-4 has become increasingly prevalent in some southern European countries on the Mediterranean Sea, with prevalence rates ranging between 7.4% and 13.2% [5][6][7][8].

The HCV-4 incidence in Greece has been reported to be 13.2% with a temporal pattern of moderate increase. Subtype 4a is the most common (78%) and the phylogenetic comparison of Greek 4a isolates has not revealed any discrimination from the HCV-4a isolates reported worldwide [7].

Fibrosis progression in HCV-4 infection has been assessed in longitudinal studies [9][10][11]. One of these comparing French, Egyptian and Sub-Saharan African HCV patients revealed significantly higher grading and staging scores in Egyptian patients infected with HCV-4a [11]. It has been hypothesized that these higher scores might be attributed to the concomitant schistosomiasis in the patient subpopulations reported above.

Because conventional interferon alpha (IFN-a) monotherapy has resulted in disappointing virologic responses, HCV-4 has been considered difficult to treat [12][13][14]. Recent reports show that the combination of pegylated interferon a (PEG-IFN-a) with ribavirin markedly improves therapeutic outcomes, resulting in sustained virologic response (SVR) rates ranging between 44% and 69% [15][16][17][18].

Despite the data reported above, the management strategies for patients infected with HCV-4 are not yet well established. The limited distribution of this genotype in Western countries and subsequently the small percentage of HCV-4 patients in major multicenter HCV therapeutic trials may in part explain this phenomenon.

On the other hand, very little is known about the influence of ethnic origin on the treatment outcome in this patient population.

## Materials and Methods

From 2003 to 2007, 60 treatment-naïve chronic HCV-4a adult patients, in total, were referred to the Gastroenterology-Hepatology and Internal Medicine-Hepatology departments of three tertiary care hospitals in Athens, Greece, for the management of hepatitis C.

Patients were stratified into two groups based on their ethnicity. Group A consisted of 30 consecutively eligible consenting European patients (24 males, 6 females), and group B included 30 consecutively eligible consenting Egyptian patients (27 males, 3 females). All patients were treated with PEG-IFN-α2a (Pegasys; Roche, Basel, Switzerland) 180 μg/wk administered subcutaneously in combination with oral ribavirin (Copegus 200 mg tablets; Roche) in a dose of 1000 or 1200 mg/day depending on baseline body weight ≤ or > 85 Kg, respectively, divided into two doses, for a period of 48 weeks.

### End Points

The primary end point of the present open-label non-interventional study was to compare the SVR rates between two ethnic groups (Europeans and Egyptians) of chronic HCV-4a infected patients with elevated HCV RNA pretreatment levels. Both groups were treated with the combination of PEG-IFN with ribavirin for a period of 48 weeks. Secondary end points were to determine the evaluation of rapid virologic response (RVR), early virologic response (EVR), and 24 week HCV RNA negativity as prognostic factors of SVR in the two studied populations. Additionally, evaluation of the safety of the treatment reported above with respect to depression and abnormal laboratory values was also performed. SVR was defined as having undetectable levels of HCV RNA 24 weeks after the EoT (week 72).

To the best of our knowledge a direct comparative study between chronic HCV-4a Europeans and Egyptians with high pretreatment HCV RNA levels, taking into account virologic response rates, has not been reported in the literature.

Patients were eligible for the study only if they were infected with genotype 4, subtype A, hepatitis C virus and they presented with HCV levels (Versant HCV RNA 3,0, Bayer Diagnostics LLC , Tarrytown, NY, USA ) > 800000 IU/ml during the previous months before enrollment in the study. Other inclusion criteria were testing anti-HCV positive, and liver biopsy performed within 6 months before treatment.

Exclusion criteria for both groups were: ethnic origin other than European or Egyptian, severe or untreated psychiatric illness, decompensated liver cirrhosis, coinfection with hepatitis B or human immunodeficiency virus or the presence of schistosoma mansoni, chronic alcohol abuse, pregnancy or lactation, severe, difficult-to-treat cardiac or neurologic disease, insulin-treated diabetes mellitus (due to the negative influence of insulin resistance on antiviral therapy), hepatocellular carcinoma (HCC) evaluated by ultrasound and alpha fetoprotein level, prior history of any other malignancy or active malignant disease, autoimmune disorders, previous treatment with IFN-α, hemoglobin level < 120g/L in women and < 130 g/L in men, a neutrophil count <1500/ mm3 and a platelet count < 75000/mm3 .

The study was approved by the ethics committees of all of the hospitals involved. All patients signed an informed consent for the collection of their data prior to participation.

In the present study, the standard therapy duration was 48 weeks, during which the two groups were treated with the dosing regimens described above.

During the 72-week study period, both groups were monitored on a monthly basis by hepatologists from the three hospitals including: physical examination, hematological and liver biochemistry tests, thyroid hormone levels measured at weeks 12, 24 and 48 and HCV RNA levels measured at weeks 4, 12, 24, 48 and 72. A liver biopsy and an HCV genotype determination were performed for all patients, prior to treatment.

For all patients, RVR and EVR to combination therapy were defined as HCV RNA levels <600 IU/ ml at weeks 4 and 12. SVR to interferon-based therapy was defined as an HCV RNA <600 IU/ml at 24 weeks post treatment.

The use of paracetamol was preferred in treating common flu-like symptoms of PEG-IFN-α2a. Dose modifications of PEG-IFN-α2a and ribavirin for adverse events and laboratory abnormalities were performed in a stepwise manner. The weekly dose of PEG-IFN-α2a could be decreased in a sequential manner in 45μg decrements from 180 μg to 135 μg to 90 μg. The daily dosage of ribavirin was to be decreased by 200 mg/day in any patient experiencing a decrease in hemoglobin level lower than 85 g/L. If the hemoglobin level dropped below this level, the administration of ribavirin was stopped.

Erythropoietin was not prescribed because the drug has no indication in Greece for anemia due to interferon or ribavirin treatment.

### Statistic analysis

Chi-squared tests with continuity correction were employed to investigate the association between the categorical variables. The homogeneity of the two groups with respect to age and body weight was tested using t-tests, whereas the Mann-Whitney U-test was employed to test differences regarding stage and grade. Statistical significance was set at α=0.05. Statistical analysis was performed using SAS 9.1.

## Results

Because all patients fulfilled the inclusion and exclusion criteria there was no significant somatic or psychiatric comorbidity. Baseline characteristics including: sex, body weight, viral load, histologic grade of liver disease, alanine aminotransferase (ALT), gamma glutamyl transferase (γGT), hemoglobin levels, white cell and platelet counts were similar in the two studied groups. However, patients from group A presented with significantly older age than those from group B (48.5±9.6, 40.93±10.2; P = 0,005) and patients from group B presented significantly higher mean ISHAK histologic stage of liver disease (2.4 ±1.4, 4.3±1.7; P < 0.001) (Table 1)

**Table 1:** Mean baseline characteristics of study groups.

**Parameter**	**Europeans (A)**	**Egyptians (B)**	**P-value**
**Gender(male/female)**	24/6	27/3	1.00
**Age(years)^a^**	48.5±9.6	40.93±10.2	0.01
**Weight(Kg)^a^**	73.03±13.4	76.00±16.3	0.45
**BMI^a^**	24.12±2.11	24.27±2.15	0.90
**Stage(Ishak)**	2.4 ±1.43	4.4 ±1.78	<0.001
**Grade^b^**	7.00 [5.75-8.00]	8.00 [5.75-9.00]	0.09
**HCV RNA**	1.5x10^6^	1.58x10^6^	0.76
**ALT (U/L) (N/A)^c^**	1/29	0/30	1.00
**γGT(U/L) (N/A)^c^**	16/14	8/22	0.06
**Hb (g/dL) (N/A)^c^**	30/0	30/0	-
**WBC ( /μ L) (N/A)^c^**	30/0	30/0	-
**PLT (x1000/ μ L) (N/A)^c^**	30/0	30/0	-

^a^ mean ±sd

^b^ median [interquartile range]

^c^ normal/abnormal

All patients from both groups completed the 48-week therapy; also all patients were followed up successfully until week 72.

Group A achieved an RVR of 26.7% (8/30), with an SVR of 62.5% (5/8). Group B achieved an RVR of 30.0% (9/30), with an SVR of 11.1% (1/9).

EVR was achieved in 23.3% (7/30) of the patients from group A with an SVR of 57.1% (4/7), and in 16.6% (5/30) from group B, with an SVR of 40% (2/5).

Week 24 negativity was 13.3% (4/30) with an SVR of 50% (2/4) in group A, and 16.6% (5/30) with an SVR of 100% (5/5) for group B.

Finally, the HCV RNA negativity at week 72 (SVR) was as follows: Group A, 11 (36.7%) patients and group B, 8 (26.7%) patients (P = 0.59) (Table 2)

**Table 2:** Initial and sustained viral response rates a.

** HCV RNA Viral Response To Treatment **					
** SVR (Week 72) **	** RR **	** No Response **	** Response **	** Group **	** Week **
0.63 (5 of 8)	0.27	22	8		4
0.57 (4 of 7)	0.23	23	7	A	12
0.5 (2 of 4)	0.13	26	4		24
0.11(1of 9)	0.3	21	9		4
0.4 (2 of 5)	0.17	25	5	B	12
1.0 (5 of 5)	0.17	25	5		24

^a^ Response Rate

**Figure 1:**
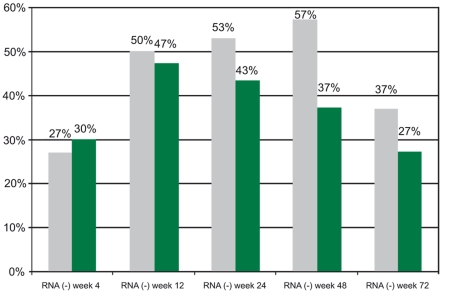
Overall HCV RNA negativity rates in both groups

Fig. 1 illustrates the overall HCV RNA negativity rates in both groups during the weeks 4, 12, 24, 48 and 72.

### Safety, use of antidepressants and abnormal laboratory values

There were no life-threatening adverse events during the study and no patients withdrew from the treatment because of a severe adverse event or laboratory abnormality.

Dose reduction of ribavirin was made in 5 (16.7%) patients from group A, and 2 (6.7%) from group B. Dose reduction of PEG-IFN-α2a was made in 4 (13.3%) patients from group A, and 3 (10%) from group B. Antidepressants (mitrazapine) were prescribed to 4 (13.3%) patients from group A.

No statistically significant percentage of abnormal hemoglobin, white blood cell, neutrophil, and platelet count values were noted in the two studied groups during the treatment period (data not shown). Patients from group B presented significantly higher percentages of abnormal ALT and γGT values at week 24 (50.0% vs. 20.0% P = 0.03; 40.0% vs. 13.3% P = 0.04) (Data not shown).

## Discussion

Very little is known about the influence of ethnic origin on the treatment outcomes in patients chronically infected with HCV-4 [4]. Results from clinical trials conducted in the Middle East and Egypt, using PEG-IFN plus ribavirin, indicated SVR rates ranging between 65% and 69% [15][16][18][19][20]. On the other hand, there are very few similar studies conducted in Europe, and none in the U.S.A. [21][22][23][24]. The vast majority of these included very small numbers of non-Sub-Saharan African and non-Egyptian patients, while others included individuals coinfected with HIV. SVR rates ranging between 16.7% and 79% have been reported from these studies.

A retrospective analysis of SVR rates among 205 naïve French and Egyptian HCV-4 patients revealed a better overall response in Egyptians than in Europeans (54.9% vs. 40.3%) treated with PEG-IFN and ribavirin [11].

In our study, the RVR and overall SVR rates (week 72) were similar in the two groups, but the manner in which overall SVR was achieved was different. In the European group, RVR was the best indicator of SVR (63%), with the lowest SVR rate (50%) in those who attained HCV RNA negativity at week 24. For the Egyptian group, RVR was the weakest indicator of SVR (11%), and HCV RNA negativity at week 24 presented the best SVR (100%) (Table 2)

The failure of the subjects at week 4 to achieve a similar SVR in the Egyptian group, after having had a similar RVR rate to the European group, along with the knowledge that the overall SVR was similar, suggests the possibility of a delay in the maintenance of treatment effect in the Egyptian population. The fact that both groups eventually do achieve similar overall SVR, keeping in mind the increased EVR and 100% SVR of the week 24 Egyptian group, strongly suggests that Egyptian patients respond to the PEG-IFN-α2a and ribavirin similarly to the European group, after the delay period passes. The possibility of a treatment delay, a type of bias present in patients with previous schistosomiasis infection due to the long-lasting liver fibrosis which it may cause, has been suggested by previous research [11]

Whether the delay in treatment response is related to the high incidence of previous schistosomiasis or to physiological differences in the Egyptian group in this study is not clear.

Although our SVR results are lower than the ones reported by Roulot et al. (36.7% vs. 40.3% for the European patients and 26.7% vs. 54.9% for the Egyptians), in a quite similar total number of HCV-4a-infected individuals, the statistical difference of SVR rates between the two populations is not significant in either of the studies. In comparing our results for the European group with those of a recent Austrian study, with a completely different study design, a lower EVR rate was observed (23.3% vs. 42.4%), but information concerning the ethnicity of patients and also the viral subtype and the baseline viral load of those who achieved EVR was not provided [25]. On the other hand, the small number of patients [9] with a viral load >400000 IU/ ml who achieved SVR makes any direct comparison with our results difficult.

Analysis of virologic response rates in the European group at weeks 4, 12 and 24 revealed that RVR was the most likely predictor of SVR (63% 5/8), while for Egyptian patients, HCV RNA negativity at week 24 was the best indicator of SVR (100% 5/5) (Table 2).

EVR can be considered a moderate prognostic factor for SVR for our European population (57%, 4/7), with a lower percentage for Egyptians (40%, 2/5). RVR rates among patients with SVR from both treated groups were as follows: 45.4%-5/11 for group A and 11.1%-1/9 for group B (Table 2). Although is difficult to provide a complete explanation for this difference, the small number of patients in the study and the advanced stage in liver histology of the Egyptian population (P < 0.001), due probably to the younger age of contamination, may provide a partial explanation. The presence of insulin resistance, which was not included in our study, may also affect the differences in viral kinetics between the two studied groups [26][27]. The expression of the MxA gene at the beginning of treatment may also be an important prognostic factor for SVR in chronic HCV-4-infected patients [28][29].

These results are different from the ones reported by Ferenci et al. for HCV-4- infected patients with baseline HCV RNA >800000 IU/ml, but the number of patients on which the conclusion is based, in the study reported above, is quite small (6 individuals) [25].

Studies of Egyptian and Saudi Arabian HCV-4- infected individuals with elevated baseline HCV RNA have reported higher SVR rates (65.0% and 42.9% respectively) than those achieved in the present study [15][17]. Although the genetic distance and heterogenecity between quasispecies can play a critical role as a predictor of IFN-based response, the unclear percentage of HCV-4a individuals and the unknown number of patients with high pretreatment viral load who achieved SVR make any direct comparison with our results difficult [26].

In a recent study, Kamal et al., have examined the significance of RVR and EVR as predictors for SVR in a group of HCV-4-infected Egyptians for a treatment duration of 48 weeks, and reported an RVR rate of 14% and an EVR rate of 26% for those who achieved SVR [19]. Although the limitations reported above still remain, our results concerning the Egyptian population are in agreement with these findings.

## Conclusions

The direct comparison between European and Egyptian HCV-4a-infected individuals with elevated baseline viral load treated with PEG-IFN-α2a and ribavirin for 48 weeks showed a higher SVR rate for the European population. RVR was a good indicator of SVR in the European group, while for the Egyptians 24 week HCV RNA negativity provided the best prognostic factor for SVR. Overall SVR was similar in both groups. The proposed therapeutic approach was well tolerated by all patients. Further studies are needed to validate our results and also to evaluate possible application to other HCV-4 groups. The individualization of HCV treatment for Egyptian patients, and the possibility of a change in the therapeutic algorithm for all genotype 4 patients should also receive further evaluation.
